# Performance of Different Risk Indicators in a Multi-Period Polynomial Portfolio Selection Problem Based on the Credibility Measure

**DOI:** 10.3390/e21050491

**Published:** 2019-05-13

**Authors:** Jian Zhou, Jie Shen, Ziheng Zhao, Yujie Gu, Mingxuan Zhao

**Affiliations:** 1 School of Management, Shanghai University, Shanghai 200444, China; 2 School of Economics, Shanghai University of Finance and Economics, Shanghai 200433, China; 3 School of Economics and Management, Tongji University, Shanghai 200082, China

**Keywords:** multi-period portfolio selection, genetic algorithm, credibility measure, risk indicator

## Abstract

In this paper, we study the portfolio selection problem considering transaction costs under multiple periods. For non-professional investors, it is a critical factor to choose an appropriate model among multiple portfolio selection models in investment. Based on the credibility measure, we formulate a multi-period polynomial portfolio selection model to gather the risk indicators involving variance, semi-variance, entropy, and semi-entropy, helping investors bet on assets. According to the polynomial goal programming (PGP) approach, investors can conquer the fields by combining apposite indicators to build appropriate models. Subsequently, an adjusted genetic algorithm on the foundation of the penalty function is designed to obtain the optimal solution of this multi-period model. The results indicate that the PGP method is suitable for investors to choose the model and assigns the proper models to investors with different risk preferences.

## 1. Introduction

Portfolio selection theory is an important part of modern quantitative finance. Generally, investors allocate the funds among different assets to maximize expected returns by choosing an optimal investment strategy, which is known as the portfolio selection problem. Since the classical portfolio selection programming was raised by Markowitz [1], who proposed the mean-variance model in which return was quantified as the mean and risk as the variance, there have been numerous studies surrounding Markowitz’s work. As research continues to deepen, new models involving other risk indicators have been introduced and enriched the research field. For example, Ballestero [2] presented mean-semi-variance in place of mean-variance owing to the fact that investors actually merely consider the risk when the returns are lower than their expected returns. Macedo et al. [3] was devoted to studying the application of multi-objective evolutionary algorithms in the portfolio optimization, within a mean-semi-variance framework, which took into account adverse return variations rather than overall variations. Meanwhile, transaction cost is also an important factor in portfolios, which has been studied by many scholars. For example, Beraldi et al. [4] aimed at dealing with complex transaction costs within portfolio management models effectively. With the increase of experts’ research on financial market, the vagueness and ambiguity of information were increasingly recognized, and the fuzzy set theory raised by Zadeh [5] was then stretched into portfolio selections. Since then, a paradigm shift has taken place while modeling portfolios, and many scholars have studied the fuzzy portfolio to deal with the uncertainty in actual problems, such as [6,7], depending on the possibility measure. Furthermore, Liu and Liu [8] transformed the exact historical data into fuzzy numbers and constrained fuzzy expected value models on the basis of the credibility measure, which is consistent with the vagueness, so as to overcome the limitations of possibility measure. In accordance with the credibility measure, Huang [9] originally put forward semi-variance to express fuzzy variance and utilized a fuzzy simulation based on the genetic algorithm to solve the formed mean-semi-variance model.

In probability theory, many researchers have investigated and accepted mean-entropy models [10,11] and mean-cross entropy models [10]; the former maximizes the uncertainty of investment return for improving diversification, and the latter minimizes the divergence of investment return from the a priori one. In fuzzy set theory, different from the probabilistic environment, Huang [12] used entropy as a measure of risk and proposed two credibility-based fuzzy mean-entropy models to illustrate the effect of entropy in portfolios. Furthermore, cross-entropy was adopted by Qin et al. [13] in portfolio problems, and a hybrid intelligent algorithm was designed to deal with different models. With the development of research, models and applications involving entropy have received great attention. For example, Ray and Majumder [14] proposed a fuzzy mean-variance-skewness-entropy model with triangular fuzzy returns to facilitate a more reasonable investment decision. Zhou et al. [15] found that semi-entropy was positively correlated with entropy and variance so that the semi-entropy can be directly proven as a surrogate indicator of risk, and an illustrative example showed that the mean-semi-entropy models of fuzzy portfolios can significantly improve the dispersibility. Tian et al. [16] established the bi-objective optimization model on the basis of fuzzy cross-entropy so as to identify the criterion weights and obtain a final ranking result.

Though great progress has been made in embedding fuzzy programming approaches in portfolios, most research on fuzzy portfolios is in the framework of a single period, and also, some scholars have studied the multi-period fuzzy portfolio selection problems. In the real world, investors tend to invest their assets from time to time, which makes it reasonable for single-period portfolio selection problems to evolve into multi-period portfolio selection problems. Yan et al. [17] originally formulated a class of multi-period semi-variance models, and a hybrid genetic algorithm was applied to solve the model. Moreover, Yan and Li [18] established a multi-period semi-variance model considering the stochastic exchange rate, which was proven to be effective. Peng et al. [19] proposed an asset-liability mean-variance model, using a nested mean-variance game formulation due to its time inconsistency. Within the framework of credibility theory, Guo et al. [20] designed a fuzzy simulation-based genetic algorithm to settle the fuzzy multi-period mean-variance model involving V-shaped transaction costs. Similarly, Zhang and Liu [21] presented a multi-period credibilistic mean-variance model, aiming to maximize the terminal wealth and minimize its risk. Besides, Mohebbi and Najaf [22] formulated a bi-objective mean-VaR portfolio selection model with multiple periods and transaction cost considerations based on credibility theory and adopted an interactive dynamic programming method to solve this model.

Table 1 summaries the literature mentioned above from the aspect of risk indicators, objective, period, and theory considered in modeling. Most of them were committed to researching one certain aspect, such as going into depth for a risk indicator, discussing multiple objectives, and studying multiple cycles. Considering that the current research mainly focuses on proposing new portfolio selection models composed of returns and a few risk indicators, such as the mean-variance model and the mean-variance-entropy model, few research puts so many risk indicators into one paper. Therefore, in order to study the extent to which these indicators affect the model, a portfolio selection model is proposed from the perspective of studying different risk indicators and their impacts in a multi-period multi-objective portfolio selection problem under the fuzzy environment. Because there exist many risk indicators, we choose variance, semi-variance, entropy, and semi-entropy, which are commonly discussed in portfolios. Meanwhile, for dealing with the multi-objective issues, a polynomial goal programming (PGP) was embedded. Ultimately, from the perspective of empirical data, the priorities to which investors with different risk preferences prefer different risk indicators or portfolio selection models involving different combination of risk indicators are given, helping investors make more rational decisions.

After this brief Introduction, this paper introduces the conceptions of credibility theory, i.e., credibilistic expectation, variance, semi-variance, entropy, and semi-entropy, in Section 2; then, based on the PGP method, a multi-period polynomial goal programming model is presented in Section 3, which considers transaction costs, upper and lower bounds, investment proportion, and short-sale to a large extent; in Section 4, the defuzzification portfolio selection model is solved by an adjusted genetic algorithm; followed by Section 5 and Section 6, providing an empirical application to demonstrate the results of the comparison of models and the final conclusion, respectively.

## 2. Preliminaries

In this section, we first illustrate some fundamental concepts including the expected value, variance, semi-variance, entropy, and semi-entropy in the credibility theory, which is the theoretical basis of this paper. Considering that much literature on fuzzy portfolios has adopted trapezoidal fuzzy numbers to characterize the fuzzy returns, such as [20,23], we then introduce some crisp expressions about indicators of trapezoidal fuzzy numbers based on the credibility measure, which is also called defuzzification. Ultimately, according to the linear property of the arithmetic operation on trapezoidal fuzzy numbers, the expressions of these indicators for the portfolio are obtained.

Let ξ be a fuzzy variable with the membership function μ. The fuzzy event *A* has the possibility [24] and the necessity [25] as:

(1) $$\mathrm{Pos}\left\{A\right\}=\underset{x\in A}{\sup}\;\mu \left(x\right),\;\mathrm{Nec}\left\{A\right\}=1-\underset{x\notin A}{\sup}\;\mu \left(x\right).$$

Referring to Liu and Liu [8], the credibility of *A* is defined by:

(2) $$\mathrm{Cr}\left\{A\right\}=\frac{1}{2}\left(\mathrm{Pos}\{A\}+\mathrm{Nec}\{A\}\right).$$

**Definition** **1.** *(Liu and Liu [8]) Let ξ be a fuzzy variable. Then, its expected value is defined by:*

(3) $$E\left[\xi \right]=\int_{0}^{+\infty }\mathrm{Cr}\left\{\xi \geq r\right\}\;dr-\int_{-\infty }^{0}\mathrm{Cr}\left\{\xi \leq r\right\}\;dr,$$

*provided that at least one of the two integrals is finite.*

**Example** **1.** *Suppose that $\xi ∼T(\underline{z},\bar{z},\delta ,\eta )$ is a trapezoidal fuzzy number, and its membership function is:*

(4) $$\mu \left(x\right)=\left\{\begin{array}{cc} \frac{x-(\underline{z}-\delta )}{\delta }, & \mathrm{if}\;\;\underline{z}-\delta \leq x<\underline{z}\\ 1, & \mathrm{if}\;\;\underline{z}\leq x\leq \bar{z}\\ \frac{(\bar{z}+\eta )-x}{\eta }, & \mathrm{if}\;\;\bar{z}<x\leq \bar{z}+\eta \\ 0, & \mathrm{otherwise}. \end{array}\right.$$

*In light of Equations (1)–(3), the expected value of ξ is (see also Liu [26]):*

(5) $$E\left[\xi \right]=\frac{2\underline{z}+2\bar{z}-\delta +\eta }{4}.$$

*Furthermore, suppose that $\xi_{i}∼T\left({\underline{z}}_{i},{\bar{z}}_{i},\delta_{i},\eta_{i}\right)$, and $x_{i}$ are nonnegative real numbers, i=1,2,…,N, representing the fuzzy return and proportion of the $i^{\mathrm{th}}$ asset, respectively. According to the linear property of the arithmetic operation on trapezoidal fuzzy numbers, it is easy to derive that:*

(6) $$\begin{array}{c} \sum_{i=1}^{N}\xi_{i}x_{i}∼T\left(\sum_{i=1}^{N}{\underline{z}}_{i}x_{i},\sum_{i=1}^{N}{\bar{z}}_{i}x_{i},\sum_{i=1}^{N}\delta_{i}x_{i},\sum_{i=1}^{N}\eta_{i}x_{i}\right). \end{array}$$

*Thus, the expected value of a portfolio is:*

(7) $$\begin{array}{c} E[\sum_{i=1}^{N}\xi_{i}x_{i}]=\frac{\sum_{i=1}^{N}\left(2{\underline{z}}_{i}+2{\bar{z}}_{i}-\delta_{i}+\eta_{i}\right)x_{i}}{4}. \end{array}$$

**Definition** **2.** *(Liu [26]) Let ξ be a fuzzy variable with a finite expected value e. Then, its variance is defined as:*

(8) $$V\left[\xi \right]=E\left[{\left(\xi -e\right)}^{2}\right].$$

**Example** **2.** *The variance of $\xi ∼T(\underline{z},\bar{z},\delta ,\eta )$ is (see also Zhang et al. [27]):*

(9) $$V\left[\xi \right]=\frac{4\epsilon^{2}+3\epsilon \theta +\theta^{2}+9\epsilon \tau +3\theta \tau +6\tau^{2}}{48}+\frac{{\left[{\left(\epsilon -\theta -2\tau \right)}^{+}\right]}^{3}}{384\epsilon },$$

*where $\epsilon =\max\left\{\delta ,\eta \right\},\;\theta =\min\left\{\delta ,\eta \right\},\;\tau =\bar{z}-\underline{z}$, and ${\left(\epsilon -\theta -2\tau \right)}^{+}=\max\left\{\epsilon -\theta -2\tau ,0\right\}$. Then, it follows from Equations (6) and (9) that the variance of a portfolio is:*

(10) $$\begin{array}{c} V\left[\sum_{i=1}^{N}\xi_{i}x_{i}\right]=\frac{4{\epsilon^{\prime }}^{2}+3\epsilon^{\prime }\theta^{\prime }+{\theta^{\prime }}^{2}+9\epsilon^{\prime }\tau^{\prime }+3\theta^{\prime }\tau^{\prime }+6{\tau^{\prime }}^{2}}{48}+\frac{{\left[{\left(\epsilon^{\prime }-\theta^{\prime }-2\tau^{\prime }\right)}^{+}\right]}^{3}}{384\epsilon^{\prime }}, \end{array}$$

*where*$\epsilon^{\prime }=\max\left\{\sum_{i=1}^{N}\delta_{i}x_{i},\sum_{i=1}^{N}\eta_{i}x_{i}\right\},\;\theta^{\prime }=\min\left\{\sum_{i=1}^{N}\delta_{i}x_{i},\sum_{i=1}^{N}\eta_{i}x_{i}\right\},\;\tau^{\prime }=\sum_{i=1}^{N}\left({\bar{z}}_{i}-{\underline{z}}_{i}\right)x_{i}$, and ${\left(\epsilon^{\prime }-\theta^{\prime }-2\tau^{\prime }\right)}^{+}=\max\left\{\epsilon^{\prime }-\theta^{\prime }-2\tau^{\prime },0\right\}$*.*

**Definition** **3.** *(Huang [9]) Let ξ be a fuzzy variable with a finite expected value e. Then, its semi-variance is defined by:*

(11) $$S_{v}\left[\xi \right]=E\left[{\left[{\left(\xi -e\right)}^{-}\right]}^{2}\right]=\int_{0}^{+\infty }\mathrm{Cr}\left\{{\left[{\left(\xi -e\right)}^{-}\right]}^{2}\geq r\right\}\;dr,$$

*where:*

(12) $${\left(\xi -e\right)}^{-}=\left\{\begin{array}{cc} \xi -e, & \mathrm{if}\;\;\xi \leq e\\ 0, & \mathrm{if}\;\;\xi >e. \end{array}\right.$$

**Example** **3.** *Suppose that $\xi ∼T(\underline{z},\bar{z},\delta ,\eta )$ and its expected value is e. The semi-variance of ξ is (see also Qian and Yin [28]):*

(13) $$S_{v}\left[\xi \right]=\left\{\begin{array}{cc} \frac{{\left(e-\underline{z}+\delta \right)}^{3}}{6\delta }, & \mathrm{if}\;\;\underline{z}-\delta \leq e<\underline{z}\\ \frac{\left(3e-3\underline{z}+\delta \right)\delta +3{\left(e-\underline{z}\right)}^{2}}{6}, & \mathrm{if}\;\;\underline{z}\leq e\leq \bar{z}\\ \frac{\left(3e-3\underline{z}+\delta \right)\delta +3\left(\bar{z}-\underline{z}\right)\left(2e-\underline{z}-\bar{z}\right)}{6}+\frac{{\left(\bar{z}-e\right)}^{2}\left(3\eta -\bar{z}+e\right)}{6\eta }, & \mathrm{if}\;\;\bar{z}<e\leq \bar{z}+\eta . \end{array}\right.$$

*Assuming that $\xi_{i}∼T\left({\underline{z}}_{i},{\bar{z}}_{i},\delta_{i},\eta_{i}\right)$ and their expected values are represented as $e_{i}$, i=1,2,…,N, then according to Equations (6) and (11)–(13), the semi-variance of a portfolio is:*

(14) $$S_{v}[\sum_{i=1}^{N}\xi_{i}x_{i}]=\left\{\begin{array}{c} \frac{{\left(\sum_{i=1}^{N}e_{i}x_{i}-\sum_{i=1}^{N}{\underline{z}}_{i}x_{i}+\sum_{i=1}^{N}\delta_{i}x_{i}\right)}^{3}}{6\sum_{i=1}^{N}\delta_{i}x_{i}},\\ \;\;\;\;\;\;\;\;\;\;\;\;\;\;\;\;\;\;\;\;\;\;\;\;\;\;\;\;\;\;\;\;\;\;\;\;\;\;\;\;\;\;\;\;\;\;\;\;\;\;\;\;\;\;\;\;\;\;\;\;\;\;\;\;\;\;\;\;\;\;\;\;\;\;\;\;\;\;\;\;\;\;\;\;\;\;\;\;\;\;\;\;\;\;\;\;\mathrm{if}\;\;\sum_{i=1}^{N}{\underline{z}}_{i}x_{i}-\sum_{i=1}^{N}\delta_{i}x_{i}\leq \sum_{i=1}^{N}e_{i}x_{i}<\sum_{i=1}^{N}{\underline{z}}_{i}x_{i}\\ \frac{1}{6}\sum_{i=1}^{N}\delta_{i}x_{i}\left(3\sum_{i=1}^{N}e_{i}x_{i}-3\sum_{i=1}^{N}{\underline{z}}_{i}x_{i}+\sum_{i=1}^{N}\delta_{i}x_{i}\right)+\frac{1}{2}{\left(\sum_{i=1}^{N}e_{i}x_{i}-\sum_{i=1}^{N}{\underline{z}}_{i}x_{i}\right)}^{2},\\ \;\;\;\;\;\;\;\;\;\;\;\;\;\;\;\;\;\;\;\;\;\;\;\;\;\;\;\;\;\;\;\;\;\;\;\;\;\;\;\;\;\;\;\;\;\;\;\;\;\;\;\;\;\;\;\;\;\;\;\;\;\;\;\;\;\;\;\;\;\;\;\;\;\;\;\;\;\;\;\;\;\;\;\;\;\;\;\;\;\;\;\;\;\;\;\;\mathrm{if}\;\;\sum_{i=1}^{N}{\underline{z}}_{i}x_{i}\leq \sum_{i=1}^{N}e_{i}x_{i}\leq \sum_{i=1}^{N}{\bar{z}}_{i}x_{i}\\ \frac{1}{6}\sum_{i=1}^{N}\delta_{i}x_{i}\left(3\sum_{i=1}^{N}e_{i}x_{i}-3\sum_{i=1}^{N}{\underline{z}}_{i}x_{i}+\sum_{i=1}^{N}\delta_{i}x_{i}\right)\\ +\frac{1}{2}\left(\sum_{i=1}^{N}{\bar{z}}_{i}x_{i}-\sum_{i=1}^{N}{\underline{z}}_{i}x_{i}\right)\left(2\sum_{i=1}^{N}e_{i}x_{i}-\sum_{i=1}^{N}{\underline{z}}_{i}x_{i}-\sum_{i=1}^{N}{\bar{z}}_{i}x_{i}\right)\\ +\frac{{\left(\sum_{i=1}^{N}{\bar{z}}_{i}x_{i}-\sum_{i=1}^{N}e_{i}x_{i}\right)}^{2}\left(3\sum_{i=1}^{N}\eta_{i}x_{i}-\sum_{i=1}^{N}{\bar{z}}_{i}x_{i}+\sum_{i=1}^{N}e_{i}x_{i}\right)}{6\sum_{i=1}^{N}\eta_{i}x_{i}},\\ \;\;\;\;\;\;\;\;\;\;\;\;\;\;\;\;\;\;\;\;\;\;\;\;\;\;\;\;\;\;\;\;\;\;\;\;\;\;\;\;\;\;\;\;\;\;\;\;\;\;\;\;\;\;\;\;\;\;\;\;\;\;\;\;\;\;\;\;\;\;\;\;\;\;\;\;\;\;\;\;\;\;\;\;\;\;\;\;\;\;\;\;\;\;\;\;\mathrm{if}\;\;\sum_{i=1}^{N}{\bar{z}}_{i}x_{i}<\sum_{i=1}^{N}e_{i}x_{i}\leq \sum_{i=1}^{N}{\bar{z}}_{i}x_{i}+\sum_{i=1}^{N}\eta_{i}x_{i}. \end{array}\right.$$

**Definition** **4.** *(Li and Liu [29]) Let ξ be a continuous fuzzy variable. Then, its entropy is defined as:*

(15) $$H\left[\xi \right]=\int_{-\infty }^{+\infty }S\left(\mathrm{Cr}\{\xi =x\}\right)\;dx,$$

*where S(t)=−tlnt−(1−t)ln(1−t) is a continuous and differentiable function, and Cr{ξ=x} is called the credibility function of ξ.*

**Example** **4.** *The entropy of $\xi ∼T(\underline{z},\bar{z},\delta ,\eta )$ is (see also Zhou et al. [30]):*

(16) $$\;H\left[\xi \right]=\frac{\eta +\delta }{2}+\left(\bar{z}-\underline{z}\right)\ln2.$$

*Then, according to Equations (6), (15) and (16), the entropy of a portfolio is:*

(17) $$\begin{array}{c} H\left[\sum_{i=1}^{N}\xi_{i}x_{i}\right]=\frac{1}{2}\sum_{i=1}^{N}\left(\eta_{i}+\delta_{i}\right)x_{i}+\ln2\sum_{i=1}^{N}\left({\bar{z}}_{i}-{\underline{z}}_{i}\right)x_{i}. \end{array}$$

**Definition** **5.** *(Zhou et al. [15]) Let ξ be a continuous fuzzy variable with a finite expected value e. Then, its semi-entropy is defined by:*

(18) $$S_{h}\left[\xi \right]=\int_{-\infty }^{+\infty }S\left(\mathrm{Cr}{\left\{\xi =x\right\}}^{-}\right)\;dx,$$

*where S(t)=−tlnt−(1−t)ln(1−t) and:*

(19) $$\mathrm{Cr}{\left\{\xi =x\right\}}^{-}=\left\{\begin{array}{cc} \mathrm{Cr}\{\xi =x\}, & \mathrm{if}\;\;\xi \leq e\\ 0, & \mathrm{if}\;\;\xi >e. \end{array}\right.$$

Since S(0)=0, the semi-entropy defined in Equation (19) can be rewritten as:

(20) $$S_{h}\left[\xi \right]=\int_{-\infty }^{e}S\left(\mathrm{Cr}\{\xi =x\}\right)\;dx.$$

**Example** **5.** *Suppose that $\xi ∼T(\underline{z},\bar{z},\delta ,\eta )$, and its expected value is e. Then, the semi-entropy of ξ is (see also Zhou et al. [15]):*

(21) $$S_{h}\left[\xi \right]=\left\{\begin{array}{cc} \delta \rho -\zeta (\rho ), & \mathrm{if}\;\;\underline{z}-\delta \leq e<\underline{z}\\ \frac{2\delta +(2\bar{z}-2\underline{z}-\delta +\eta )\ln2}{4}, & \mathrm{if}\;\;\underline{z}\leq e\leq \bar{z}\\ \frac{\delta }{2}+\left(\bar{z}-\underline{z}\right)\ln2+\eta (\zeta \left(\sigma \right)-\sigma +\frac{1}{2}), & \mathrm{if}\;\;\bar{z}<e\leq \bar{z}+\eta , \end{array}\right.$$

$where\;\rho =\left(2\bar{z}-2\underline{z}+3\delta +\eta \right)/8\delta ,\;\sigma =\left(2\bar{z}-2\underline{z}+\delta +3\eta \right)/8\eta ,\;and\;\zeta \left(x\right)=x^{2}\ln x-{\left(1-x\right)}^{2}\ln\left(1-x\right).$ *Assuming that $\xi_{i}∼T\left({\underline{z}}_{i},{\bar{z}}_{i},\delta_{i},\eta_{i}\right)$ and their expected values are represented as $e_{i}$, i=1,2,…,N, then according to Equations (6), (20) and (21), the semi-entropy of a portfolio is:*

(22) $$S_{h}[\sum_{i=1}^{N}\xi_{i}x_{i}]=\left\{\begin{array}{c} \rho^{\prime }\sum_{i=1}^{N}\delta_{i}x_{i}-\zeta \left(\rho^{\prime }\right),\\ \;\;\;\;\;\;\;\;\;\;\;\;\;\;\;\;\;\;\;\;\;\;\;\;\;\;\;\;\;\;\;\;\;\;\;\;\;\;\;\;\;\;\;\;\;\;\;\;\;\;\;\;\;\;\;\;\;\;\;\;\;\;\;\;\;\;\;\;\;\;\;\;\;\;\;\;\;\;\;\;\;\;\;\;\;\;\;\;\;\;\;\;\;\;\;\;\mathrm{if}\;\;\sum_{i=1}^{N}{\underline{z}}_{i}x_{i}-\sum_{i=1}^{N}\delta_{i}x_{i}\leq \sum_{i=1}^{N}e_{i}x_{i}<\sum_{i=1}^{N}{\underline{z}}_{i}x_{i}\\ \frac{\ln2}{4}\left(2\sum_{i=1}^{N}{\bar{z}}_{i}x_{i}-2\sum_{i=1}^{N}{\underline{z}}_{i}x_{i}-\sum_{i=1}^{N}\delta_{i}x_{i}+\sum_{i=1}^{N}\eta_{i}x_{i}\right)+\frac{1}{2}\sum_{i=1}^{N}\delta_{i}x_{i},\\ \;\;\;\;\;\;\;\;\;\;\;\;\;\;\;\;\;\;\;\;\;\;\;\;\;\;\;\;\;\;\;\;\;\;\;\;\;\;\;\;\;\;\;\;\;\;\;\;\;\;\;\;\;\;\;\;\;\;\;\;\;\;\;\;\;\;\;\;\;\;\;\;\;\;\;\;\;\;\;\;\;\;\;\;\;\;\;\;\;\;\;\;\;\;\;\;\mathrm{if}\;\;\sum_{i=1}^{N}{\underline{z}}_{i}x_{i}\leq \sum_{i=1}^{N}e_{i}x_{i}\leq \sum_{i=1}^{N}{\bar{z}}_{i}x_{i}\\ \frac{1}{2}\sum_{i=1}^{N}\delta_{i}x_{i}+\left(\sum_{i=1}^{N}{\bar{z}}_{i}x_{i}-\sum_{i=1}^{N}{\underline{z}}_{i}x_{i}\right)\ln2+\sum_{i=1}^{N}\eta_{i}x_{i}\left(\zeta \left(\sigma^{\prime }\right)-\sigma^{\prime }+\frac{1}{2}\right),\\ \;\;\;\;\;\;\;\;\;\;\;\;\;\;\;\;\;\;\;\;\;\;\;\;\;\;\;\;\;\;\;\;\;\;\;\;\;\;\;\;\;\;\;\;\;\;\;\;\;\;\;\;\;\;\;\;\;\;\;\;\;\;\;\;\;\;\;\;\;\;\;\;\;\;\;\;\;\;\;\;\;\;\;\;\;\;\;\;\;\;\;\;\;\;\;\;\mathrm{if}\;\;\sum_{i=1}^{N}{\bar{z}}_{i}x_{i}<\sum_{i=1}^{N}e_{i}x_{i}\leq \sum_{i=1}^{N}{\bar{z}}_{i}x_{i}+\sum_{i=1}^{N}\eta_{i}x_{i}, \end{array}\right.$$

$where\;\rho^{\prime }=\left(2\sum_{i=1}^{N}{\bar{z}}_{i}x_{i}-2\sum_{i=1}^{N}{\underline{z}}_{i}x_{i}+3\sum_{i=1}^{N}\delta_{i}x_{i}+\sum_{i=1}^{N}\eta_{i}x_{i}\right)/8\sum_{i=1}^{N}\delta_{i}x_{i},\;and\;\sigma^{\prime }=\left(2\sum_{i=1}^{N}{\bar{z}}_{i}x_{i}-2\sum_{i=1}^{N}{\underline{z}}_{i}x_{i}+\sum_{i=1}^{N}\delta_{i}x_{i}+3\sum_{i=1}^{N}\eta_{i}x_{i}\right)/8\sum_{i=1}^{N}\eta_{i}x_{i}.$

## 3. Portfolio Selection Models

In this section, considering that constructing novel portfolio selection models is a highly valued work and studied by many scholars, we are committed to studying the performance of different risk indicators in models from a completely different perspective. For this purpose, a fuzzy multi-period polynomial goal programming model considering transaction costs is proposed. In this polynomial model, the effects of different risk indicators can be observed by changing the parameters of the objective function. For better understanding, the variables and parameters used in this model are first described in Table 2, and the return of each asset is assumed to be a trapezoidal fuzzy number.

### 3.1. The Multi-Period Polynomial Goal Programming Model

Considering the complexity of the market environment, we measure risk from different aspects to construct portfolio selection models to help investors with different risk preferences. In order to deal with the multi-objective problem, we introduce the PGP method mentioned in [31], transforming multiple targets into a single target, and further effectively compare the different risk indicators. Specifically, we apply the defuzzification approach introduced in Section 2 to obtain crisp forms of objective functions.

First of all, a multi-period multi-objective portfolio selection model P(1) can be constructed by taking both returns and risks into account, as follows,

(23) $$P\left(1\right)\left\{\begin{array}{c} \max\;\;O_{1}=\prod_{t=1}^{T}\left[1+E\left[\sum_{i=1}^{N}\xi_{i}x_{it}\right]-\sum_{i=1}^{N}c_{it}|x_{it}-x_{i(t-1)}|\right]-1\\ \min\;\;O_{2}=\sum_{t=1}^{T}V\left[\sum_{i=1}^{N}\xi_{i}x_{it}\right]\\ \min\;\;O_{3}=\sum_{t=1}^{T}S_{v}\left[\sum_{i=1}^{N}\xi_{i}x_{it}\right]\\ \min\;\;O_{4}=\sum_{t=1}^{T}H\left[\sum_{i=1}^{N}\xi_{i}x_{it}\right]\\ \min\;\;O_{5}=\sum_{t=1}^{T}S_{h}\left[\sum_{i=1}^{N}\xi_{i}x_{it}\right]\\ \mathrm{subject}\;\mathrm{to}:\\ \;\;\;\;\;\;\;\;\;\;\;\;\sum_{i=1}^{N}x_{it}=1,\;t=1,2,\ldots ,T\\ \;\;\;\;\;\;\;\;\;\;\;\;l_{it}y_{it}\leq x_{it}\leq u_{it}y_{it},\;i=1,2,\ldots ,N,\;t=1,2,\ldots ,T\\ \;\;\;\;\;\;\;\;\;\;\;\;x_{it}\geq 0,\;i=1,2,\ldots ,N,\;t=1,2,\ldots ,T\\ \;\;\;\;\;\;\;\;\;\;\;\;y_{it}\in \left\{0,\;1\right\},\;i=1,2,\ldots ,N,\;t=1,2,\ldots ,T. \end{array}\right.$$

As can be observed above, the functions $O_{2}$–$O_{5}$ are separately minimizing the variance, semi-variance, entropy, and semi-entropy of a portfolio during the whole investment period, corresponding to Equations (5), (10), (14), (17), and (22). The function $O_{1}$ is aimed at maximizing the overall expected return of a portfolio excluding transaction costs throughout the whole period, which can be derived by the following process. Firstly, we can easily get the total wealth at period *T*,

(24) $$\begin{array}{cc} W_{T} & =W_{T-1}\left[1+E\left[\sum_{i=1}^{N}\xi_{i}x_{it}\right]-\sum_{i=1}^{N}c_{it}|x_{iT}-x_{i(T-1)}|\right]\\ & =W_{0}\prod_{t=1}^{T}\left[1+E\left[\sum_{i=1}^{N}\xi_{i}x_{it}\right]-\sum_{i=1}^{N}c_{it}|x_{it}-x_{i(t-1)}|\right]. \end{array}$$

Obviously, the overall expected return of a portfolio at period *T* is:

(25) $$\begin{array}{cc} R_{T} & =\frac{W_{0}\prod_{t=1}^{T}\left[1+E\left[\sum_{i=1}^{N}\xi_{i}x_{it}\right]-\sum_{i=1}^{N}c_{it}|x_{it}-x_{i(t-1)}|\right]-W_{0}}{W_{0}}\\ & =\prod_{t=1}^{T}\left[1+E\left[\sum_{i=1}^{N}\xi_{i}x_{it}\right]-\sum_{i=1}^{N}c_{it}|x_{it}-x_{i(t-1)}|\right]-1. \end{array}$$

As for the constraints, the first one ensures that all funds are invested in each period. The second constraint limits the proportion of each asset that is held to the lower and upper limits in each period, and the proportion is zero if the asset is not held. The third constraint means no short-selling of assets. Moreover, the last constraint is the integrality constraint.

In order to solve this complex multi-objective model, the PGP method is implemented. In the PGP process, there are two steps. Firstly, we must concentrate on solving each individual objective function to achieve aspired levels $R^{*},\;V^{*},\;S_{v}^{*},\;H^{*}$, and $S_{h}^{*}$. For example, $R^{*}$ can be obtained by solving the sub-individual objective model consisting of $O_{1}$ and all the constraints.

In the second step, goal variables $d_{1},\;d_{2},\;d_{3},\;d_{4}$, and $d_{5}$ ($d_{i}\geq 0$) are employed to depict the deviations from aspired levels. Meanwhile, the Minkowski distance is commonly used to transform multiple objectives into a single target model. Similar to Lai [32], first normalize the Minkowski distance, and then, we can get a standardized expression. Furthermore, with reference to Aksarayli and Pala [31], we added one to all normalized goals, guaranteeing the base number of each objective to be greater than one, so that the goal variables can increase strictly with the exponent. Then, considering the investors’ preferences for the model, $\lambda_{i}$ was utilized to prioritize the five targets. For different values of $\lambda_{i}$, the optimal values in different scenarios can be obtained.

On the basis of the PGP approach described above, the multi-objective model P(1) can be transformed into the polynomial model P(2), which is constructed as:

(26) $$P\left(2\right)\left\{\begin{array}{c} \min\;\;Z={\left(1+|\frac{d_{1}}{R^{*}}|\right)}^{\lambda_{1}}+{\left(1+|\frac{d_{2}}{V^{*}}|\right)}^{\lambda_{2}}+{\left(1+|\frac{d_{3}}{S_{v}^{*}}|\right)}^{\lambda_{3}}+{\left(1+|\frac{d_{4}}{H^{*}}|\right)}^{\lambda_{4}}+{\left(1+|\frac{d_{5}}{S_{h}^{*}}|\right)}^{\lambda_{5}}\\ \mathrm{subject}\;\mathrm{to}:\\ \;\;\;\;\;\;\;\;\;\;\;\;\;\prod_{t=1}^{T}\left[1+E\left[\sum_{i=1}^{N}\xi_{i}x_{it}\right]-\sum_{i=1}^{N}c_{it}|x_{it}-x_{i(t-1)}|\right]-1+d_{1}=R^{*}\\ \;\;\;\;\;\;\;\;\;\;\;\;\;\sum_{t=1}^{T}V\left[\sum_{i=1}^{N}\xi_{i}x_{it}\right]-d_{2}=V^{*}\\ \;\;\;\;\;\;\;\;\;\;\;\;\;\sum_{t=1}^{T}S_{v}\left[\sum_{i=1}^{N}\xi_{i}x_{it}\right]-d_{3}=S_{v}^{*}\\ \;\;\;\;\;\;\;\;\;\;\;\;\;\sum_{t=1}^{T}H\left[\sum_{i=1}^{N}\xi_{i}x_{it}\right]-d_{4}=H^{*}\\ \;\;\;\;\;\;\;\;\;\;\;\;\;\sum_{t=1}^{T}S_{h}\left[\sum_{i=1}^{N}\xi_{i}x_{it}\right]-d_{5}=S_{h}^{*}\\ \;\;\;\;\;\;\;\;\;\;\;\;\;\sum_{i=1}^{N}x_{it}=1,\;t=1,2,\ldots ,T\\ \;\;\;\;\;\;\;\;\;\;\;\;\;l_{it}y_{it}\leq x_{it}\leq u_{it}y_{it},\;i=1,2,\ldots ,N,\;t=1,2,\ldots ,T\\ \;\;\;\;\;\;\;\;\;\;\;\;\;x_{it}\geq 0,\;i=1,2,\ldots ,N,\;t=1,2,\ldots ,T\\ \;\;\;\;\;\;\;\;\;\;\;\;\;y_{it}\in \left\{0,\;1\right\},\;i=1,2,\ldots ,N,\;t=1,2,\ldots ,T. \end{array}\right.$$

In the model P(2), $\lambda_{i}$ measures the degree of an investor’s preference: the larger $\lambda_{i}$ is, the more important the corresponding indicator is. Meanwhile, it is worth noting that risk is always positive, and thus, the objective can be simplified to:

$$Z={\left(1+|\frac{d_{1}}{R^{*}}|\right)}^{\lambda_{1}}+{\left(1+\frac{d_{2}}{V^{*}}\right)}^{\lambda_{2}}+{\left(1+\frac{d_{3}}{S_{v}^{*}}\right)}^{\lambda_{3}}+{\left(1+\frac{d_{4}}{H^{*}}\right)}^{\lambda_{4}}+{\left(1+\frac{d_{5}}{S_{h}^{*}}\right)}^{\lambda_{5}}.$$

For the purpose of better comparing the impact of different risk indicators on the models, $\lambda_{1}$ was fixed to one, and $\lambda_{2}$–$\lambda_{5}$ were binary variables in the empirical experiments of this paper, resulting in different models with different risk indicators. For example, $\lambda_{1}$ = $\lambda_{2}$ = 1 and $\lambda_{3}$ = $\lambda_{4}$ = $\lambda_{5}$ = 0 mean only the overall expected return and variance of a portfolio work in this model.

Thus, these commonly-used risk indicators can be embedded into one objective function through the PGP method, then the impact of risk indicators on the models can be studied by adjusting the value of the parameters, which is the biggest difference between this paper and other literature.

### 3.2. Portfolio Performance Measures

In order to evaluate the performance of different models, the Sharpe ratio, a traditional performance measure, was utilized. On the basis of credibility theory, we adopted the credibilistic Sharpe ratio (CrSR) presented by Jalota et al. [23] to measure risk-adjusted return, specifically the expected fuzzy return divided by the fuzzy standard deviation. Here, aiming to optimize the overall multi-period portfolio, the CrSR can be computed as:

(27) $$\mathrm{CrSR}=\frac{\prod_{t=1}^{T}\left[1+E\left[\sum_{i=1}^{N}\xi_{i}x_{it}\right]-\sum_{i=1}^{N}c_{it}|x_{it}-x_{i(t-1)}|\right]-1}{\sqrt{\sum_{t=1}^{T}V\left[\sum_{i=1}^{N}\xi_{i}x_{it}\right]}}.$$

This indicator was utilized to assess the performance of portfolio selection models by considering the benefits and risks in a comprehensive manner. A larger number indicates a higher return per unit risk.

Besides, similar to Aksarayli and Pala [31] and DeMiguel and Nogales [33], we compared portfolio models with average portfolio turnover (PT) as a value of the magnitude of the transaction cost in different periods, which is computed as:

(28) $$\mathrm{PT}=\frac{1}{T}\sum_{t=1}^{T}\sum_{i=1}^{n}|x_{it}-x_{i(t-1)}|.$$

In short, two completely different measures, CrSR and PT, were introduced to better evaluate the different models in the empirical experiments so that the analysis results can be more objective and fair.

## 4. Adjusted Genetic Algorithm

In this section, the adjusted genetic algorithm proposed in this study will be described in detail. Especially, the algorithm was run six times independently, for which the previous five runs were in parallel, corresponding to the five sub-individual objective models, and the aspired levels $R^{*},\;V^{*},\;S_{v}^{*},\;H^{*}$, and $S_{h}^{*}$ can be separately obtained. Then, through embedding these aspired levels into the last one, the optimal solution of the final polynomial model P(2) can be achieved. The variables and parameters used in this section are introduced in Table 3.

### 4.1. Initialization

Denote the population size of chromosomes as *n*. Each chromosome is a *T*×*N* matrix, which can be expressed as:

$$X_{c}=\left[x_{c1};x_{c2};\ldots ;x_{cT}\right]=\left[\begin{array}{cccc} x_{c1}^{1} & x_{c1}^{2} & \ldots & x_{c1}^{N}\\ x_{c2}^{1} & x_{c2}^{2} & \ldots & x_{c2}^{N}\\ \vdots & \vdots & \ddots & \vdots \\ x_{cT}^{1} & x_{cT}^{2} & \ldots & x_{cT}^{N} \end{array}\right],\;c=1,2,\ldots ,n,$$

representing the $c^{\mathrm{th}}$ portfolio. Each element, $x_{ct}^{i}$, represents the proportion of the $i^{\mathrm{th}}$ asset of the $t^{\mathrm{th}}$ period of the $c^{\mathrm{th}}$ portfolio, which is a non-negative value generated randomly, and each row can be expressed as:

$$x_{ct}=\left(x_{ct}^{1},x_{ct}^{2},\ldots ,x_{ct}^{N}\right),\;c=1,2,\ldots ,n,\;t=1,2,\ldots ,T,$$

representing the $t^{\mathrm{th}}$ period of the $c^{\mathrm{th}}$ portfolio. Besides, through normalization, the sum of the proportion of investment at each period was forced to be one, that is,

(29) $$\sum_{i=1}^{N}x_{ct}^{i}=1,\;c=1,2,\ldots ,n,\;t=1,2,\ldots ,T.$$

### 4.2. Crossover

Denote the probability of crossover as mc. Only if a uniformly-distributed random number generated was smaller than mc, we conducted the crossover process. In this process, traditionally, the parents are usually two different chromosomes. Here, considering the need to control the overall cost of the portfolio, it was necessary to reduce the turnover. For this purpose, we respectively regarded the $t^{\mathrm{th}}$ and [t+T/2]^th^ ([t+T/2] means down round) rows of the selected chromosome $X_{c}$ as parents, that is $x_{ct}$ and $x_{c[t+T/2]}$. For t=1,2,…,T/2, the crossover process is shown as:

$$x_{ct}^{\prime }=x_{ct}+\gamma_{1}|x_{c[t+T/2]}-x_{ct}|,$$

$$x_{c[t+T/2]}^{\prime }=x_{c[t+T/2]}+\gamma_{2}|x_{c[t+T/2]}-x_{ct}|.$$

Here, $\gamma_{1}$ and $\gamma_{2}$ are generated randomly, whose values range from (0,1). The selected chromosome is transformed into:

$$X_{c}^{\prime }=\left[x_{c1}^{\prime };x_{c2}^{\prime };\ldots ;x_{cT}^{\prime }\right],\;c=1,2,\ldots ,n.$$

### 4.3. Mutation

Denote the probability of mutation as mu. Only if a uniformly-distributed random number generated was smaller than mu, we conducted the mutation process. Similar to the traditional approach, some genes were randomly selected from the chromosome for mutation. In order to mutate this matrix chromosome, for each row, $x_{ct}\;\left(t=1,2,\ldots ,T\right)$, we randomly picked the $i^{\mathrm{th}}$ element and replaced it with the (*i*+1)^th^ element of another row, $x_{ct^{*}}\;\left(t\neq t^{*}\right)$. Meanwhile, the (*i*+1)^th^ element of $x_{ct}$ was replaced by the $i^{\mathrm{th}}$ element of $x_{ct*}$. The above mutation process is shown as:

$${\left(x_{ct}^{i}\right)}^{\prime }=x_{ct^{*}}^{i+1},$$

$${\left(x_{ct}^{i+1}\right)}^{\prime }=x_{ct^{*}}^{i}.\;$$

After the crossover and mutation, the current chromosomes may not satisfy the constraint in Equation (29) that the sum of the proportion of investment per period needs to be one, so the renormalization was performed.

### 4.4. Evaluation

Traditionally, we utilize the objective function as the search information during the evaluation process in the genetic algorithm. Because there exist multiple constraints, we applied the self-adapting penalty coefficient C(κ) into the selection instead. C(κ) is defined as:

$$C\left(\kappa \right)=10^{1-\kappa }-1,$$

$$\kappa =\frac{m}{n},\;$$

where *m* is the number of feasible solutions that meet all the constraints and *n* is the population size.

Since *κ* is the ratio of *m* to *n*, it also symbolizes the probability. There are few feasible solutions in the early stages of the iterations, so the penalty coefficient was large, narrowing the gap between chromosomes and the feasible region. With the iterations conducted, there were more solutions in the later stages of iterations, so the self-adapting penalty coefficient became relatively small, focusing more on finding the optimal solution.

We then define the evaluation function $G_{u}\left(x\right)\;\left(u=1,2,\ldots ,6\right)$ as:

$$G_{u}\left(x\right)=f_{u}\left(x\right)+C\left(\kappa \right)*\left(\varphi_{1}\left(x\right)+\varphi_{2}\left(x\right)\right),$$

in which:

$$\begin{array}{c} f_{1}\left(x\right)=\prod_{t=1}^{T}\left[1+E\left[\sum_{i=1}^{N}\xi_{i}x_{it}\right]-\sum_{i=1}^{N}c_{it}|x_{it}-x_{i(t-1)}|\right]-1,\\ f_{2}\left(x\right)=\sum_{t=1}^{T}V\left[\sum_{i=1}^{N}\xi_{i}x_{it}\right],\\ f_{3}\left(x\right)=\sum_{t=1}^{T}S_{v}\left[\sum_{i=1}^{N}\xi_{i}x_{it}\right],\\ f_{4}\left(x\right)=\sum_{t=1}^{T}H\left[\sum_{i=1}^{N}\xi_{i}x_{it}\right],\\ f_{5}\left(x\right)=\sum_{t=1}^{T}S_{h}\left[\sum_{i=1}^{N}\xi_{i}x_{it}\right],\\ f_{6}\left(x\right)={\left(1+|\frac{d_{1}}{R^{*}}|\right)}^{\lambda_{1}}+{\left(1+\frac{d_{2}}{V^{*}}\right)}^{\lambda_{2}}+{\left(1+\frac{d_{3}}{S_{v}^{*}}\right)}^{\lambda_{3}}+{\left(1+\frac{d_{4}}{H^{*}}\right)}^{\lambda_{4}}+{\left(1+\frac{d_{5}}{S_{h}^{*}}\right)}^{\lambda_{5}},\\ C\left(\kappa \right)=10^{1-\kappa }-1,\\ \varphi_{1}\left(x\right)={\left(\max\{0,l_{i}y_{i}-x_{i}\}\right)}^{2},\\ \varphi_{2}\left(x\right)={\left(\max\{0,x_{i}-u_{i}y_{i}\}\right)}^{2}. \end{array}$$

It is worth noting that experiments were separately conducted for each of the six $f_{u}\left(x\right)$. According to the implementations of the models on $f_{1}\left(x\right),f_{2}\left(x\right),\ldots ,f_{5}\left(x\right)$, we can get $R^{*},\;V^{*},\;S_{v}^{*},\;H^{*}$, and $S_{h}^{*}$, which were used to embed into $f_{6}\left(x\right)$ to obtain the final result of the portfolio selection model.

### 4.5. Selection

After the evaluation process, we achieved n processed chromosomes. By the tournament selection mentioned by Deb [34], half of the chromosomes that were better with a smaller $G_{u}\left(x\right)$ named as winners were achieved by competition. Meanwhile, all the feasible solutions were picked out by checking all the constraints. The union set of the winners and the feasible solutions contributed in subsequent iterations. Besides, some chromosomes were generated randomly to make the total number in the next iteration always equal to *n*. Finally, after *M* iterations of crossover, mutation, evaluation, and selection, the final optimal solution can be reported, where *M* is the number of generations.

## 5. Empirical Experiments

In this section, filtering all stocks of the Shanghai Stock Exchange (SSE) 50 index, 29 stocks with complete ten-year historical data (December 2007–2017) were selected on Wind (a data service software). There were two main considerations in choosing the ten-year data. On the one hand, the acquisition of fuzzy numbers was consistent. On the other hand, the long-existing stocks are safer for investors.

**Figure 1:**
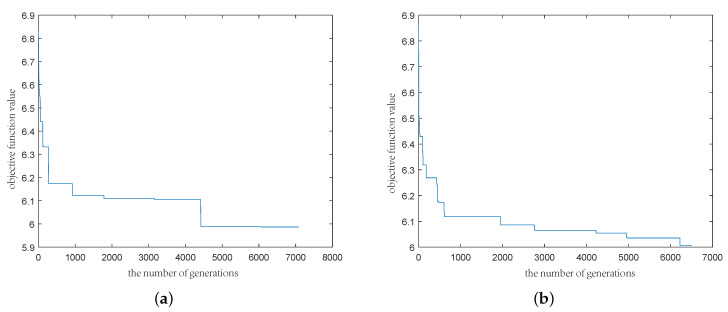
The change of the objective function value with the number of generations. (**a**) The result of the first experiment; (**b**) The result of the second experiment.

For each stock, we obtained the closing prices of 121 months. By the formula of monthly return $r_{it}=\left(P_{it+1}-P_{it}\right)/P_{it}$, we obtained 120 historical yield rates, which were sorted by reference to Bermúdez et al. [35]. The 40–60 sample percentiles of the monthly returns were regarded as the cores of each fuzzy return, while the 5–40 and 60–95 sample percentiles expressed the left-hand and right-hand spreads of each trapezoidal fuzzy number. Hence, 29 trapezoidal fuzzy variables were achieved (see Table 4). Furthermore, in the ensuing demonstration, the constant parameters in the models were adopted as: $l_{it}=0$, $u_{it}=0.2$, $c_{it}=0.03\;\left(i=1,2,\ldots ,29;\;t=1,2,\ldots ,12\right)$. By using the software MATLAB, we set the parameters of the designed algorithm as: the population size was n=500, the crossover probability mc=0.9, the mutation probability mu=0.1, and the number of generations M= 10,000. With regard to the polynomial model corresponding to T=12, $\lambda_{1}=\lambda_{2}=\lambda_{5}=1$, and $\lambda_{3}=\lambda_{4}=0$, Figure 1a,b show how the objective function value changes with the number of generations. The first experiment converges around 4000 and the second converges around 6000. Thus, in order to ensure the reliability of the results, we set M= 10,000.

Table 5 provides the aspired values $R^{*},V^{*},S_{v}^{*},H^{*}$, and $S_{h}^{*}$, which were obtained by separately handling the five sub-individual objective models. Because of too many periods and experimental data, in order to be brief, we just show the results of the last three issues of the whole 12 periods in the tables.

We embedded the aspired values in the objective and constraints of the P(2) model to settle the models involving different combinations of objective functions. Simultaneously, two groups were constructed by assigning a binary value (0,1) to the corresponding $\lambda_{i}$ (i = 2, 3, 4, 5) so as to analyze the performance of different risk indicators. Specifically, Group 1 consisted of the overall expected return and a risk indicator, including the return-variance model (RVM), RS_*v*_M, RHM, and RS_*h*_M, respectively; the corresponding $\lambda_{i}$ were 1-1-0-0-0, 1-0-1-0-0, 1-0-0-1-0, and 1-0-0-0-1. Group 2 consisted of the overall expected return and two risk indicators, including the return-variance-entropy model (RVHM), RVS_*h*_M, RS_*v*_HM, and RS_*v*_S_*h*_M, respectively; the corresponding $\lambda_{i}$ were 1-1-0-1-0, 1-1-0-0-1, 1-0-1-1-0, and 1-0-1-0-1.

Table 6 presents the results of each objective value of the models in Group 1 in Periods 10–12. By observing the results of Periods 10–12, comparing RVM and RS_*v*_M, the variance, semi-variance, entropy, and semi-entropy of the former were smaller, indicating that the risk was smaller, while the latter had better expected return. According to the comprehensive CrSR index, the RS_*v*_M model was better. In addition, comparing RHM and RS_*h*_M, the variance, semi-variance, entropy, and semi-entropy of the latter were smaller, showing that the risk was smaller, while the former had better expected return. The comprehensive CrSR index expressed that the RHM model was better. Overall, the largest expected return was obtained by RHM, followed by RS_*h*_M; relatively speaking, they were income preferences. For the remaining risk indicators including variance, semi-variance, entropy, and semi-entropy, RVM had the lowest risk and RS_*v*_M the second, indicating that they were risk aversions. Here, we used the indicator CrSR to balance the return and risk, and the results showed that RHM was the best, followed by RS_*h*_M, suggesting that the return had a greater contribution to the CrSR.

Table 7 presents the results of each objective value of the models in Group 2 in Periods 10–12. By observing the results of Periods 10–12, comparing RVHM and RVS_*h*_M, the variance, semi-variance, entropy, and semi-entropy of the latter were smaller, indicating that the risk was smaller, while the former had better expected return. According to the comprehensive CrSR index, the RVHM model was better. In addition, comparing RS_*v*_HM and RS_*v*_S_*h*_M, the former had better expected return, and its risk was relatively smaller in terms of probability; thus, the comprehensive CrSR index was obviously better. Compare the models RVHM and RS_*v*_HM, which both contain the entropy issue, and models RVS_*h*_M and RS_*v*_S_*h*_M, which both contain the semi-entropy issue; the semi-variance issue prefers the expected return, while the variance issue contributes greatly to avoiding risk. Overall, the largest expected return was obtained by RS_*v*_HM, followed by RS_*v*_S_*h*_M; relatively speaking, they were income preferences. For the remaining risk indicators including variance, semi-variance, entropy, and semi-entropy, RVS_*h*_M had the lowest risk, and RVHM was the second, indicating that they were risk aversions. Here, we used the indicator CrSR to balance the return and risk, and the results showed that RS_*v*_HM was the best, followed by RS_*v*_S_*h*_M.

**Figure 2:**
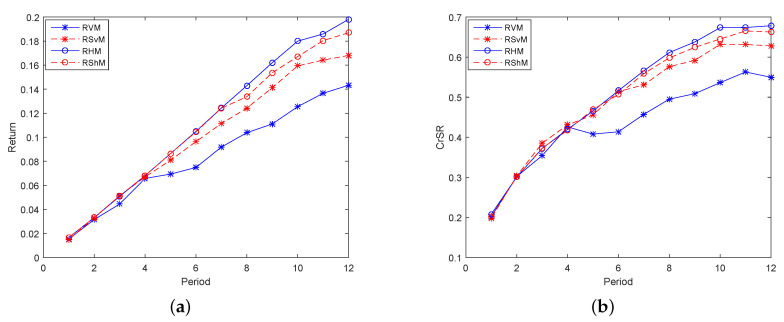
The performance of the models in Group 1 in terms of return in 12 periods. (**a**) The expected returns of the models in Group 1 in 12 periods; (**b**) The CrSRs of the models in Group 1 in 12 periods.

**Figure 3:**
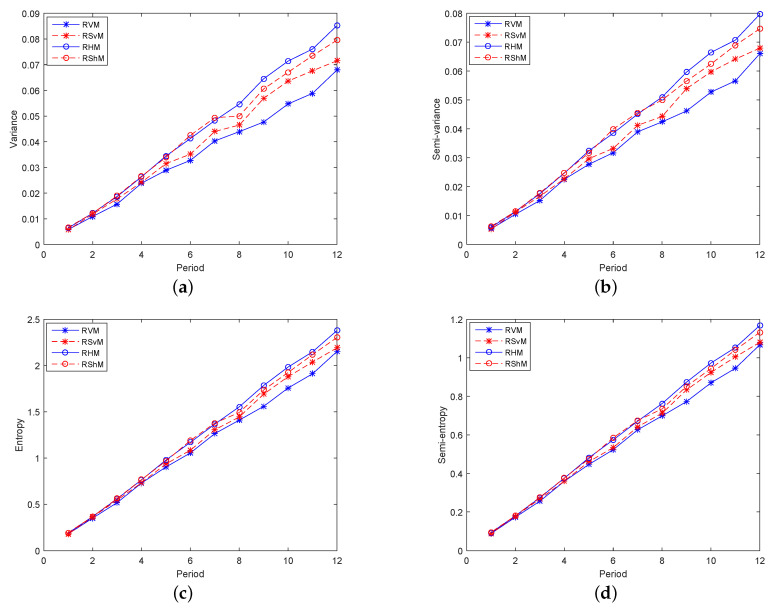
The performance of the models in Group 1 in terms of risk in 12 periods. (**a**) The variances of the models in Group 1 in 12 periods; (**b**) The semi-variances of the models in Group 1 in 12 periods; (**c**) The entropies of the models in Group 1 in 12 periods; (**d**) The semi-entropies of the models in Group 1 in 12 periods.

To better illustrate the above analysis, we conduct a study of each indicator in the 12 periods. Firstly, study models consisting of two indicators, one return indicator and one risk indicator, made up Group 1. Figure 2a,b, respectively, shows the expected returns and CrSRs of models in Group 1 in 12 periods, and their trends were basically the same. For investors who prefer incomes, the larger the expected returns and CrSRs are, the better the model is. Obviously, entropy plays the most important role, followed by semi-entropy. Therefore, for risk-averse investors, the four risk indicators with basically consistent trends in Figure 3a–d shows that the contribution of variance was the largest, followed by semi-variance. Hence, for investors who balance incomes and risks, RHM was chosen in the high-yield models, and RS_*v*_M was chosen in the low-risk models because their corresponding CrSRs were relatively higher.

To sum up, for the models in Group 1, the four risk indicators can be divided into two categories: one is the income-preference index composed of entropy and semi-entropy, and the other one is the risk aversion index composed of variance and semi-variance. In terms of performance, entropy was the best from the perspective of profit, and the variance was optimal from the perspective of risk. In equilibrium, the entropy was optimal in the high-yield fields, and the semi-variance performed best in the low-risk fields.

**Figure 4:**
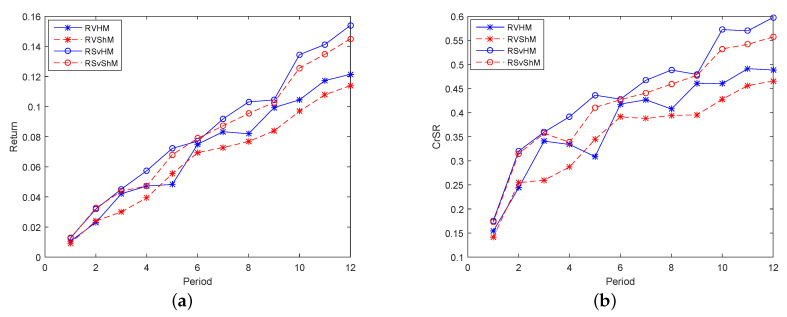
The performance of the models in Group 2 in terms of return in 12 periods. (**a**) The expected returns of the models in Group 2 in 12 periods; (**b**) The CrSRs of the models in Group 2 in 12 periods.

**Figure 5:**
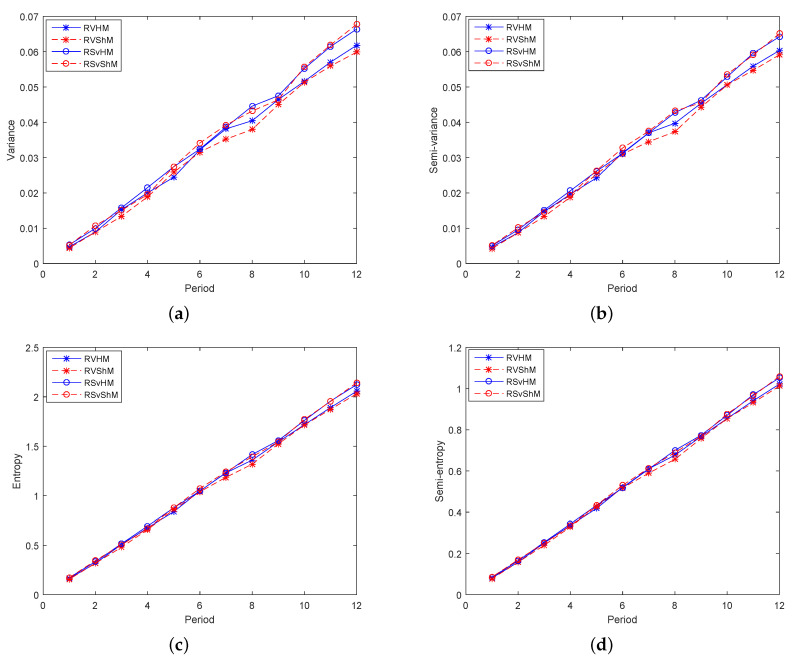
The performance of the models in Group 2 in terms of risk in 12 periods. (**a**) The variances of the models in Group 2 in 12 periods; (**b**) The semi-variances of the models in Group 2 in 12 periods; (**c**) The entropies of the models in Group 2 in 12 periods; (**d**) The semi-entropies of the models in Group 2 in 12 periods.

Secondly, models consisting of three indicators were studied, that is one return indicator and two risk indicators made up Group 2. For investors who prefer incomes, Figure 4a,b shows that RS_*v*_HM and RS_*v*_S_*h*_M were higher than RVHM and RVS_*h*_M. For models with three indicators, the semi-variance element was more helpful for expected returns and CrSRs than variance, and the model with entropy named RS_*v*_HM was the best. In addition, for investors who avoid risks, Figure 5a–d displays that the results of the four risk indicators were quite close, and the risks of RVHM and RVS_*h*_M were smaller than those of RS_*v*_HM and RS_*v*_S_*h*_M, indicating that the variance had a greater effect on risk aversions and that the model with semi-entropy named RVS_*h*_M was the best. Hence, for investors who balance incomes and risks, RS_*v*_HM was chosen in the high-yield models, and RVHM was chosen in the low-risk models because of their higher CrSRs.

To sum up, for the models in Group 2, the risks of the four models were roughly equivalent. Similarly, the four models can be divided into two categories: one is the risk-avoidance models based on variance, and the other one is the income-preference models based on semi-variance. In terms of performance, RS_*v*_HM was the optimal from the perspective of profit, and RVS_*h*_M performed best from the perspective of risk. In terms of balance, RS_*v*_HM was the best in the high-yield fields, and RVHM was optimal in the low-risk fields.

## 6. Conclusions

In this paper, we investigated the performance of different risk indicators in a multi-period polynomial portfolio selection problem considering transaction costs based on the credibility measure. For consistency, we used the PGP method to fuse the variance, semi-variance, entropy, and semi-entropy of trapezoidal fuzzy numbers obtained from historical data into the P(2) model, and an alterable new target can be constructed by the Minkowski distance and normalization methods. Then, in order to further deal with the nonlinear model, we used the penalty function to construct an adjusted genetic algorithm.

The comparison results indicated that for individual risk indicators, entropy was the best from the perspective of profit and variance was the best from the perspective of risk. In equilibrium, entropy was the winner in high-yield fields, and semi-entropy was the winner in low-risk fields. Meanwhile, for the combination of two different types of risk indicators, RS_*v*_HM was the optimal from the perspective of profit, and RVS_*h*_M performed best from the perspective of risk. In terms of balance, RS_*v*_HM was the best in the high-yield field, and RVHM was optimal in the low-risk field. In general, the participation of entropy and variance can respectively be helpful for investors with income preferences and risk avoidance. Furthermore, the classification of each model is shown in Table 8 by comparing the performances of the two groups, and we have the following conclusions for investors with different preferences. For investors seeking security and risk avoidance, RVS_*h*_M was optimal because of its lowest risk value. For investors seeking high return, RHM was the best choice for its highest overall expected return, while for investors who balance return and risk, due to the highest CrSR, RS_*v*_M was the best.

## Figures and Tables

**Table 1:** Literature on indicators related to portfolio selection problems.

Literature	Indicators								Objective		Period		Theory	
	Mean	Variance	Semi-Variance	Skewness	VaR	Entropy	Semi-Entropy	Cross-Entropy	Single	Multiple	Single	Multiple	Probability	Fuzzy
Markowitz [1]	*√*	*√*							*√*		*√*		*√*	
Ballestero [2]	*√*		*√*						*√*		*√*		*√*	
Macedo et al. [3]	*√*		*√*							*√*	*√*		*√*	
Tanaka and Guo [6]; Zhang and Nie [7]	*√*	*√*							*√*		*√*			*√*
Huang [9]	*√*		*√*						*√*		*√*			*√*
Fang et al. [10]; Ray and Majumder [11]	*√*					*√*			*√*		*√*		*√*	
Fang et al. [10]	*√*							*√*	*√*		*√*		*√*	
Huang [12]	*√*					*√*			*√*		*√*			*√*
Qin et al. [13]	*√*	*√*	*√*					*√*	*√*		*√*			*√*
Ray and Majumder [14]	*√*	*√*		*√*		*√*			*√*		*√*			*√*
Zhou et al. [15]	*√*						*√*		*√*		*√*			*√*
Tian et al. [16]	*√*							*√*		*√*	*√*			*√*
Yan et al. [17]; Yan and Li [18]	*√*		*√*						*√*			*√*	*√*	
Peng et al. [19]	*√*	*√*								*√*		*√*	*√*	
Guo et al. [20]	*√*	*√*							*√*			*√*		*√*
Zhang and Liu [21]	*√*	*√*								*√*		*√*		*√*
Mohebbi and Najaf [22]	*√*				*√*					*√*		*√*		*√*
Our Paper	*√*	*√*	*√*			*√*	*√*			*√*		*√*		*√*

**Table 2:** Notation for the portfolio selection model.

*N*	the number of stocks
*T*	the number of periods
*i*	the index of stocks, i=1,2,…,N
*t*	the index of periods, t=1,2,…,T
$x_{it}$	the proportion of the $i^{\mathrm{th}}$ asset in the portfolio at period *t*
$\xi_{i}$	the fuzzy return of the $i^{\mathrm{th}}$ asset
$c_{it}$	the unit transaction cost of the $i^{\mathrm{th}}$ asset at period *t*
$l_{it}$	the minimum proportion of the $i^{\mathrm{th}}$ asset, which is held at period *t*
$u_{it}$	the maximum proportion of the $i^{\mathrm{th}}$ asset, which is held at period *t*
$y_{it}$	a binary variable representing whether the $i^{\mathrm{th}}$ asset is held at period *t*
$W_{t}$	the wealth at period *t*
$W_{0}$	the initial wealth

**Table 3:** Notation for the adjusted genetic algorithm.

*n*	the number of chromosomes
$X_{c}$	the $c^{\mathrm{th}}$ chromosome (the $c^{\mathrm{th}}$ portfolio), c=1,2,…,n
$x_{ct}$	the $t^{\mathrm{th}}$ row of the $c^{\mathrm{th}}$ chromosome (the $t^{\mathrm{th}}$ period of the $c^{\mathrm{th}}$ portfolio), t=1,2,…,T, c=1,2,…,n
$x_{ct}^{i}$	the $i^{\mathrm{th}}$ gene of the $t^{\mathrm{th}}$ row of the $c^{\mathrm{th}}$ chromosome (the proportion of the $i^{\mathrm{th}}$ asset of the $t^{\mathrm{th}}$ period
	of the $c^{\mathrm{th}}$ portfolio), i=1,2,…,N, t=1,2,…,T, c=1,2,…,n
mc	the probability of crossover
mu	the probability of mutation
*m*	the number of feasible solutions
κ	the ratio of the number of feasible solutions to the number of chromosomes
C(κ)	the self-adapting penalty coefficient
G(x)	the evaluation function
f(x)	the objective function
φ(x)	the penalty objects
g(x)	the constraints
*M*	the number of generations

**Table 4:** Trapezoid fuzzy numbers of 29 stocks.

*n*	StockCode	$\underline{z}$	$\bar{z}$	δ	η
1	600000.SH	−0.023628034	0.021299214	0.13610887	0.174083048
2	600016.SH	−0.009861757	0.020130273	0.167089241	0.119344766
3	600019.SH	−0.019900655	0.024544972	0.195422382	0.157829398
4	600028.SH	−0.005669648	0.016583748	0.18382707	0.132929631
5	600029.SH	−0.034205738	0.040079047	0.22833473	0.246465345
6	600030.SH	−0.03245946	0.021286388	0.21818683	0.225210624
7	600036.SH	−0.014447506	0.020828916	0.152308873	0.12974701
8	600048.SH	−0.020446583	0.032400037	0.173950382	0.179271575
9	600104.SH	−0.010417911	0.045085116	0.205592922	0.154787862
10	600111.SH	−0.035568294	0.023282572	0.183286255	0.246846825
11	600309.SH	−0.016353897	0.038295577	0.168562951	0.16340411
12	600340.SH	−0.00723589	0.054566129	0.153655402	0.252402741
13	600518.SH	−0.005617978	0.045311627	0.161402331	0.192679292
14	600519.SH	−0.005361561	0.030734486	0.121306114	0.121561626
15	600547.SH	−0.020963099	0.020944349	0.192934999	0.234550977
16	600837.SH	−0.02941196	0.023945956	0.205097228	0.236746591
17	600887.SH	−0.009470846	0.046021836	0.12411694	0.134626751
18	601006.SH	−0.017600688	0.011538044	0.110527539	0.106417475
19	601088.SH	−0.020717318	0.013337128	0.152134264	0.153252715
20	601166.SH	−0.015894578	0.030576748	0.187454657	0.181398303
21	601169.SH	−0.016085791	0.02102592	0.141549217	0.145565389
22	601318.SH	−0.009346851	0.021530804	0.171072217	0.166800067
23	601328.SH	−0.015609632	0.016279685	0.15428676	0.178860513
24	601390.SH	−0.030057803	0.011279535	0.137041933	0.17993819
25	601398.SH	0	0.019288022	0.117650834	0.066160636
26	601601.SH	−0.014823329	0.02713941	0.178869489	0.180670459
27	601628.SH	−0.014412609	0.019612925	0.181438145	0.171312746
28	601857.SH	−0.011700917	0.006418605	0.154594454	0.070365245
29	601988.SH	−0.010831256	0.014392963	0.121767208	0.06468867

**Table 5:** The aspired values of objectives at the $T^{\mathrm{th}}$ period.

Period	$R^{*}$	$V^{*}$	$S_{v}^{*}$	$H^{*}$	$S_{h}^{*}$
*T*=10	0.1803	0.0382	0.0371	1.4325	0.7371
*T*=11	0.2026	0.0419	0.0407	1.5803	0.8107
*T*=12	0.2208	0.0458	0.0444	1.7156	0.8880

**Table 6:** Objective values of models in Group 1 at the $T^{\mathrm{th}}$ period. RVM, return-variance model; RVHM, return-variance-entropy model.

Period	Model	Preferences	*R*	*V*	$S_{v}$	*H*	$S_{h}$	CrSR	PT
*T* = 10	RVM	1-1-0-0-0	0.1255	0.0547	0.0527	1.7569	0.8700	0.5363	0.3797
	RS_*v*_M	1-0-1-0-0	0.1594	0.0636	0.0597	1.8790	0.9239	0.6318	0.3440
	RHM	1-0-0-1-0	0.1800	0.0714	0.0664	1.9834	0.9729	0.6735	0.3603
	RS_*h*_M	1-0-0-0-1	0.1671	0.0671	0.0625	1.9246	0.9443	0.6452	0.4304
*T* = 11	RVM	1-1-0-0-0	0.1366	0.0589	0.0566	1.9104	0.9456	0.5629	0.4406
	RS_*v*_M	1-0-1-0-0	0.1642	0.0676	0.0642	2.0374	1.0046	0.6314	0.4075
	RHM	1-0-0-1-0	0.1859	0.0761	0.0707	2.1479	1.0533	0.6740	0.3647
	RS_*h*_M	1-0-0-0-1	0.1804	0.0735	0.0690	2.1174	1.0411	0.6654	0.3503
*T* = 12	RVM	1-1-0-0-0	0.1433	0.0679	0.0660	2.1514	1.0678	0.5496	0.4145
	RS_*v*_M	1-0-1-0-0	0.1681	0.0715	0.0680	2.1905	1.0806	0.6283	0.4169
	RHM	1-0-0-1-0	0.1981	0.0852	0.0797	2.3784	1.1681	0.6784	0.4080
	RS_*h*_M	1-0-0-0-1	0.1870	0.0796	0.0746	2.3032	1.1324	0.6629	0.3927

**Table 7:** Objective values of models in Group 2 at the $T^{\mathrm{th}}$ period.

Period	Model	Preferences	*R*	*V*	$S_{v}$	*H*	$S_{h}$	CrSR	PT
*T* = 10	RVHM	1-1-0-1-0	0.1045	0.0516	0.0506	1.7179	0.8547	0.4599	0.4278
	RVS_*h*_M	1-1-0-0-1	0.0969	0.0513	0.0506	1.7149	0.8543	0.4276	0.3838
	RS_*v*_HM	1-0-1-1-0	0.1345	0.0552	0.0529	1.7606	0.8702	0.5723	0.4886
	RS_*v*_S_*h*_M	1-0-1-0-1	0.1256	0.0557	0.0536	1.7711	0.8768	0.5322	0.3487
*T* = 11	RVHM	1-1-0-1-0	0.1173	0.0570	0.0559	1.8910	0.9410	0.4912	0.4155
	RVS_*h*_M	1-1-0-0-1	0.1079	0.0559	0.0547	1.8734	0.9315	0.4561	0.3879
	RS_*v*_HM	1-0-1-1-0	0.1412	0.0615	0.0596	1.9567	0.9704	0.5697	0.3954
	RS_*v*_S_*h*_M	1-0-1-0-1	0.1347	0.0619	0.0590	1.9531	0.9645	0.5416	0.3791
*T* = 12	RVHM	1-1-0-1-0	0.1214	0.0618	0.0603	2.0562	1.0217	0.4884	0.4225
	RVS_*h*_M	1-1-0-0-1	0.1139	0.0599	0.0591	2.0304	1.0123	0.4656	0.3823
	RS_*v*_HM	1-0-1-1-0	0.1539	0.0664	0.0642	2.1224	1.0521	0.5975	0.4256
	RS_*v*_S_*h*_M	1-0-1-0-1	0.1450	0.0678	0.0651	2.1415	1.0596	0.5571	0.4202

**Table 8:** Summary of the return and risk of the models.

		Return		
		Low	Medium	High
Risk	Low	RVHM		
		RVS_*h*_M *√*		
	Medium		RVM	
			RS_*v*_M *√*	
			RS_*v*_HM	
			RS_*v*_S_*h*_M	
	High			RHM*√*
				RS_*h*_M

## Abbreviations

The following abbreviations are used in this manuscript:

PGP	polynomial goal programming
CrSR	credibilistic Sharpe ratio
PT	portfolio turnover
SSE	Shanghai Stock Exchange
